# Serum phytanic and pristanic acid levels and prostate cancer risk in Finnish smokers

**DOI:** 10.1002/cam4.319

**Published:** 2014-08-16

**Authors:** Margaret E Wright, Demetrius Albanes, Ann B Moser, Stephanie J Weinstein, Kirk Snyder, Satu Männistö, Peter H Gann

**Affiliations:** 1 Department of Pathology, College of Medicine, University of Illinois at ChicagoChicago, Illinois; 2 American Academy of PediatricsElk Grove Village, Illinois; 3 Nutritional Epidemiology Branch, Division of Cancer Epidemiology and Genetics, National Cancer Institute, NIH, DHHSBethesda, Maryland; 4 Kennedy Krieger InstituteBaltimore, Maryland; 5 Information Management Services Inc.Silver Spring, Maryland; 6 Department of Chronic Disease Prevention, National Institute for Health and WelfareHelsinki, Finland

**Keywords:** Biomarker, diet, phytanic acid, pristanic acid, prostate cancer

## Abstract

Phytanic acid is a saturated branched-chain fatty acid found predominantly in red meat and dairy products, and may contribute to the elevated risks of prostate cancer associated with higher consumption of these foods. Pristanic acid is formed during peroxisomal oxidation of phytanic acid, and is the direct substrate of *α*-Methyl-CoA-Racemase (AMACR)—an enzyme that is consistently overexpressed in prostate tumors relative to benign tissue. We measured phytanic and pristanic acids as percentages of total fatty acids by gas chromatography-mass spectrometry in prediagnostic blood samples from 300 prostate cancer cases and 300 matched controls, all of whom were participants in the Alpha-Tocopherol, Beta-Carotene Cancer Prevention (ATBC) Study supplementation trial and follow-up cohort. In addition to providing a fasting blood sample at baseline, all men completed extensive diet, lifestyle, and medical history questionnaires. Among controls, the strongest dietary correlates of serum phytanic and pristanic acids were saturated fat, dairy fat, and butter (*r* = 0.50 and 0.40, 0.46 and 0.38, and 0.40 and 0.37, respectively; all *P*-values <0.001). There was no association between serum phytanic acid and risk of total or aggressive prostate cancer in multivariate logistic regression models (for increasing quartiles, odds ratios (OR) and 95% confidence intervals (CI) for aggressive cancer were 1.0 (referent), 1.62 (0.97–2.68), 1.12 (0.66–1.90), and 1.14 (0.67–1.94), *P*_trend_ = 0.87). Pristanic acid was strongly correlated with phytanic acid levels (*r* = 0.73, *P* < 0.0001), and was similarly unrelated to prostate cancer risk. Significant interactions between phytanic and pristanic acids and baseline circulating *β*-carotene concentrations were noted in relation to total and aggressive disease among participants who did not receive *β*-carotene supplements as part of the original ATBC intervention trial. In summary, we observed no overall association between serum phytanic and pristanic acid levels and prostate cancer risk. Findings indicating that the direction and magnitude of these associations depended upon serum levels of the antioxidant *β*-carotene among men not taking *β*-carotene supplements should be interpreted cautiously, as they are likely due to chance.

## Introduction

Increased consumption of dairy products and red meat have been linked with higher risks of prostate cancer—particularly aggressive disease—in many epidemiological studies [1–6]. Much effort has been directed toward elucidating specific compounds in these foods that may be responsible for the aforementioned associations. Total and saturated fat (for dairy and red meat), calcium and protein/insulin-like growth factor (IGF)-1 (for dairy), and heme iron and heterocyclic amines (for red meat) have been intensively investigated, but none of these factors have been consistently associated with prostate cancer risk [7–9].

Phytanic acid is a saturated branched-chain fatty acid found predominantly in high-fat dairy products and red meat [10]. Ruminant animals including cows, sheep, and goats harbor bacteria in their gut that degrade chlorophyll into phytol and then phytanic acid. As humans are unable to release phytol from chlorophyll, we obtain phytanic acid exclusively from these dietary sources [11]. Phytanic acid is metabolized to pristanic acid during peroxisomal *α*-oxidation; pristanic acid is the direct substrate of *α*-Methyl-CoA-Racemase (AMACR), one of the enzymes that are crucial for proper degradation of phytanic acid. AMACR is so consistently overexpressed in prostate tumors compared to benign tissue that it is used clinically as a marker to identify prostate cancer in ambiguous biopsies [12]. Furthermore, polymorphisms in the AMACR gene have been linked with elevated risks of prostate cancer in several independent studies [13–16]. These findings highlight the potential importance of phytanic and pristanic acids—either directly or through their interaction with AMACR—in prostate carcinogenesis. Although the exact mechanism(s) remain unclear, it is possible that excessive levels of phytanic or pristanic acids could lead to overexpression of AMACR or to increased generation of reactive oxygen species-known byproducts of phytanic acid metabolism [17].

Two studies to date have investigated the association between blood levels of phytanic acid and prostate cancer risk. A small case–control study found modest yet significantly higher serum phytanic acid concentrations in prostate cancer cases versus controls [18], and a larger nested case–control study showed significant elevations in risk among men with higher fasting concentrations of plasma phytanic acid [19]. We sought to further clarify this relationship using nested case–control data from a large cohort of Finnish male smokers. We also report the first findings regarding the association between pristanic acid and prostate cancer risk.

## Materials and Methods

### Study population

The Alpha-Tocopherol, Beta-Carotene Cancer Prevention (ATBC) Study was a randomized, double-blind, placebo-controlled trial that tested whether daily supplementation with *β*-carotene (20 mg) and/or vitamin E (50 mg dL-*α*-tocopheryl acetate) reduced the incidence of lung and other cancers. Details about study design, methods, participant characteristics, and compliance have been reported [20], as have the main trial findings for selected cancers [21]. Briefly, 29,133 participants meeting all eligibility criteria at entry (male resident of southwestern Finland aged 50–69 years who smoked five or more cigarettes per day) were successfully randomized between 1985 and 1988. Reasons for exclusion included a prior history of cancer (other than nonmelanoma skin cancer or carcinoma in situ), serious illness, or refusal to discontinue use of vitamin E, vitamin A, or *β*-carotene supplements in excess of predefined amounts. Capsule compliance was high (99%) in all groups during the intervention period, and serum concentrations for *β*-carotene and *α*-tocopherol increased at 3 years by 17-fold and 50%, respectively, in the active treatment arms (no change was noted in the nontreatment arms) [22]. The randomized phase of the trial ended on 30 April 1993 after 5–8 years of active intervention (median, 6.1 years), and ascertainment of morbidity and mortality end points continued thereafter. The institutional review boards of both the National Public Health Institute of Finland (currently National Institute for Health and Welfare) and the U.S. National Cancer Institute approved the study, and written informed consent was obtained from each participant before randomization.

### Case and control selection

Incident cases of prostate cancer (ICD-9 code 185) were identified through the Finnish Cancer Registry, which provides close to 100% case ascertainment for the ATBC cohort [23]. For cases diagnosed through July 2002, medical records were reviewed centrally by one or two independent clinical oncologists for diagnostic confirmation and staging. Information on prostate cancer cases diagnosed after this point in time was derived from the Finnish Cancer Registry, with more than half also reviewed by one oncologist. We selected 300 incident cases at random from among 1345 prostate cancers diagnosed on or before 30 June 2007; we purposely overselected cases with advanced disease (*n* = 199: stage III or IV of the tumor-node-metastasis staging system, as defined by the American Joint Committee on Cancer [24], and/or those with a Gleason grade of 8 or higher). Incidence density sampling with replacement was used to randomly select controls who were alive and free of cancer at the time of a case's diagnosis. Controls were matched to cases in a 1:1 ratio on age (±5 years) and date of baseline serum blood draw (±30 days).

### Baseline data collection

Before randomization, all subjects were asked to provide detailed demographic and smoking information, to give a history of physician-confirmed diseases, and to complete a 276-item dietary questionnaire that ascertained both frequency of intake and portion size. A color picture booklet was provided to each participant in order to assist with portion size estimation. The dietary questionnaire was developed specifically for use in the ATBC trial, and was validated against food consumption records in a pilot study conducted in middle-aged Finnish men [25]. An overnight fasting blood sample was collected from virtually all participants at baseline, and serum concentrations of *α*-tocopherol, *β*-carotene, and retinol were determined using high-performance liquid chromatography [26].

### Serum phytanic and pristanic acid assays

Serum total lipid phytanic and pristanic fatty acid levels were measured as their pentafluorobenzyl bromide esters by capillary gas chromatography negative ion mass spectrometry with quantitation by selected ion monitoring in ratio to stable isotope-labeled internal standards [27]. A single measurement was used after a pilot study of fasting blood samples obtained from six males showed very little variation in phytanic and pristanic acid levels between blinded replicates. Fatty acid levels were expressed as weight-per-volume and as the weight percentage of total fatty acids; the latter units were emphasized in primary analyses. Matched cases and controls were placed adjacently within batches. Multiple quality control samples obtained from a single pool of male fasting serum were inserted randomly within each batch, and all laboratory personnel were blinded to the identity of the samples. Mean intra and interbatch coefficients of variation were 3% and 6.7% for phytanic acid and 4.5% and 8.7% for pristanic acid.

### Statistical analysis

Serum concentrations of phytanic and pristanic acids were log transformed to approximate a normal distribution. One control was identified as an outlier and excluded from all analyses. Differences in baseline characteristics were evaluated using *t*-tests for continuous variables and *χ*^2^ tests for categorical variables. Associations between serum phytanic and pristanic acids and selected dietary factors were assessed in controls using Spearman correlation coefficients both adjusted and unadjusted for total energy intake. Unconditional logistic regression models adjusted for matching factors were used to calculate odds ratios (OR) and 95% confidence intervals (CI) for prostate cancer among each quartile of serum phytanic and pristanic acids, with the lowest category serving as the referent group. Tests for linear trend were carried out by taking the median value of each quartile and modeling as a continuous variable. Addition of smoking dose and duration, trial intervention group, body mass index (BMI), education level, personal history of diabetes or benign prostatic hyperplasia, family history of prostate cancer, physical activity level, alcohol consumption, and intakes of fruit and vegetables, lycopene, *α*-tocopherol, vitamin D, or calcium to this model did not alter ORs by more than 10%.

We evaluated effect modification by trial intervention assignment (α-tocopherol, no *α*-tocopherol, *β*-carotene, no *β*-carotene), family history of prostate cancer (yes, no), and BMI (<25, ≥25 kg/m^2^), as well as by age, baseline serum levels of *α*-tocopherol and *β*-carotene, baseline dietary intakes of fruit and vegetables, carotenoids, vitamin E, and vitamin C, smoking dose and duration, and alcohol consumption. Interactions were tested for statistical significance by including cross-product terms in the appropriate multivariate models.

All analyses were conducted with SAS (version 9.2, SAS Institute Inc., Cary, NC), and statistical tests were two-tailed with significance levels set at *P* < 0.05.

## Results

Cases and controls did not differ significantly with respect to age (a matching factor), BMI, smoking dose, history of benign prostatic hyperplasia or diabetes, family history of prostate cancer, physical activity, alcohol consumption, or dietary intake of fat, saturated fat, or fruit and vegetables (Table 1). However, cases reported significantly greater attained education and shorter smoking duration, and had higher baseline serum *β*-carotene levels, than controls. Mean levels of serum phytanic and pristanic acids were similar in cases and controls.

**Table 1:** Selected baseline characteristics (mean and standard deviation, or proportion) of prostate cancer cases and controls.

Characteristic	Cases (*n* = 300)	Controls (*n* = 299)	*P*
Age (years)	57.9 (5.2)	57.8 (5.2)	0.82
Body mass index (kg/m^2^)	26.0 (3.3)	26.5 (3.6)	0.10
Cigarettes per day	19.2 (8.9)	19.5 (7.9)	0.63
Years of smoking	35.3 (8.9)	36.7 (8.0)	0.04
History of BPH (% yes)	4.7	3.0	0.29
History of diabetes (% yes)	4.0	3.0	0.51
Family history of prostate cancer (% yes)	7.6	4.9	0.21
Physical activity (% active)^1^	62.0	57.2	0.23
Education > primary school (% yes)	41.0	30.4	0.007
Serum cholesterol (mmol/L)	6.27 (1.09)	6.24 (1.30)	0.74
Serum *β*-carotene (*μ*g/L)	242 (197)	214 (130)	0.04
Serum *α*-tocopherol (mg/L)	11.8 (2.4)	11.9 (2.4)	0.87
Alcohol consumption (g/d)	16.3 (20.4)	16.7 (19.2)	0.80
Energy intake (kcal/d)	2675 (757)	2696 (819)	0.74
Fat intake (g/d)^2^	117 (15)	117 (15)	0.64
Saturated fat intake (g/d)^2^	49.7 (12.1)	50.4 (12.3)	0.50
Fruit and vegetable intake (g/d)^2^	242 (132)	241 (128)	0.94
*β*-Carotene intake (*μ*g/d)^2^	2097 (1382)	2044 (1333)	0.62
Total dairy product intake (g/d)^2^^,^^3^	780	771	0.74
Red meat intake (g/d)^2^^,^^4^	32.0	31.5	0.79
Estimated phytanic acid intake (mg/d)^2^^,^^5^	129 (54)	133 (56)	0.45
Serum phytanic acid (% total fatty acids)^6^	0.08 (0.04)	0.08 (0.04)	0.83
Serum phytanic acid (*μ*g/mL)	3.02 (1.57)	3.09 (1.77)	0.97
Serum pristanic acid (% total fatty acids)^6^	0.01 (0.007)	0.01 (0.008)	0.45
Serum pristanic acid (*μ*g/mL)	0.50 (0.32)	0.51 (0.28)	0.59

The ATBC Study. BPH, benign prostatic hyperplasia.

1 Moderate or heavy leisure activity.

2 Adjusted for energy intake.

3 Includes butter, cheese, cream, cultured milk products, ice cream, low fat and whole milk, sour milk products, and yogurt.

4 Includes beef, sausages and cold cuts, and blood, liver and inner organs; pork excluded since it does not contain any phytanic acid.

5 Described in reference [28].

6 Geometric mean concentrations.

The strongest dietary determinants of circulating levels of phytanic acid were saturated fat, dairy fat, butter fat, butter, and whole milk; red meat and fatty fish exhibited little correlation with phytanic acid (Table 2). Notably, intake of phytanic acid, whose estimation is described in reference [28], was highly correlated with serum concentrations of both phytanic and pristanic acids (*r* = 0.45 and 0.38, respectively; *P*-values <0.0001). Serum phytanic and pristanic acids were highly correlated with one another (Spearman correlation coefficient = 0.73, *P*-value <0.0001), and it is therefore not surprising that the same dietary factors were the strongest predictors of both fatty acids.

**Table 2:** Spearman correlation coefficients between serum phytanic and pristanic acids (as % of total fatty acids) and selected dietary factors in 284 controls.^1^

Dietary factor (g/day)	Phytanic acid		Pristanic acid	
	
	Partial correlation coefficient^2^	*P*	Partial correlation coefficient^2^	*P*
Total dairy products	0.20	0.0007	0.10	0.10
Butter	0.40	<0.0001	0.37	<0.0001
Cheese	0.06	0.30	0.06	0.33
Hard fatty cheese	0.07	0.25	0.05	0.37
Cream	0.11	0.05	0.13	0.03
Ice cream	−0.05	0.38	−0.05	0.43
Whole milk	0.29	<0.0001	0.27	<0.0001
Low-fat milk	−0.10	0.08	−0.11	0.08
Yogurt	−0.009	0.88	0.04	0.53
Sour milk products	0.06	0.34	0.06	0.32
Total red meat^3^	−0.03	0.62	0.03	0.67
Beef	−0.02	0.72	0.04	0.46
Lamb	0.17	0.005	0.18	0.002
Sausages	−0.14	0.02	−0.08	0.20
Fatty fish^4^	−0.004	0.95	0.07	0.27
Total fat	0.25	<0.0001	0.20	0.0006
Saturated fat	0.50	<0.0001	0.40	<0.0001
Dairy fat	0.46	<0.0001	0.38	<0.0001
Butter fat	0.40	<0.0001	0.36	<0.0001
Estimated phytanic acid intake^5^	0.45	<0.0001	0.38	<0.0001

1 Duplicate controls removed from analysis.

2 Adjusted for energy intake.

3 Includes contributions from beef, lamb, and liver and blood products.

4 Includes contributions from herring, tuna, and sardines.

5 Estimation described in reference [28].

In multivariate models, serum concentrations of phytanic and pristanic acids were unrelated to risks of overall and aggressive prostate cancer (Table 3). The ratio of pristanic to phytanic acid levels—an indicator of peroxisomal AMACR activity—was also unrelated to risk.

**Table 3:** OR and 95% CI for prostate cancer risk according to quartiles of serum phytanic and pristanic acids (as % of total fatty acids) in the ATBC Study.

	Quartiles				*P*_trend_
	
	1	2	3	4	
Phytanic acid					
Median (IQR), controls only	0.04 (0.018)	0.07 (0.015)	0.095 (0.013)	0.13 (0.021)	
*All prostate cancer cases*					
Cases/controls (*n*)	70/75	97/75	71/75	62/74	0.51
Multivariate^1^ OR (95% CI)	1.0 (ref)	1.39 (0.89–2.17)	1.01 (0.63–1.60)	0.90 (0.56–1.44)	
*Aggressive*^2^ *prostate cancer cases*					
Cases/controls (*n*)	41/75	66/75	46/75	46/74	0.87
Multivariate^1^ OR (95% CI)	1.0 (ref)	1.62 (0.97–2.68)	1.12 (0.66–1.90)	1.14 (0.67–1.94)	
Pristanic acid					
Median (IQR), controls only	0.007 (0.0030)	0.011 (0.0017)	0.015 (0.0029)	0.022 (0.0067)	
*All prostate cancer cases*					
Cases/controls (*n*)	92/75	67/75	79/75	62/74	0.15
Multivariate^1^ OR (95% CI)	1.0 (ref)	0.72 (0.46–1.13)	0.86 (0.55–1.33)	0.68 (0.43–1.08)	
*Aggressive*^2^ *prostate cancer cases*					
Cases/controls (n)	56/75	47/75	54/75	42/74	0.40
Multivariate^1^ OR (95% CI)	1.0 (ref)	0.83 (0.50–1.38)	0.96 (0.59–1.57)	0.76 (0.46–1.27)	
Pristanic/phytanic acid ratio					
Median (IQR), controls only	0.11 (0.02)	0.14 (0.02)	0.18 (0.03)	0.25 (0.06)	
*All prostate cancer cases*					
Cases/controls (*n*)	88/75	61/75	83/75	68/74	0.54
Multivariate^1^ OR (95% CI)	1.0 (ref)	0.69 (0.44–1.10)	0.95 (0.61–1.47)	0.79 (0.50–1.24)	
*Aggressive*^2^ *prostate cancer cases*					
Cases/controls (*n*)	56/75	40/75	57/75	46/74	0.80
Multivariate^1^ OR (95% CI)	1.0 (ref)	0.72 (0.43–1.20)	1.03 (0.63–1.68)	0.84 (0.50–1.40)	

95% CI, 95% confidence interval; IQR, interquartile range; OR, odds ratio.

1 Unconditional logistic regression models adjusted for age and date of serum blood draw (matching factors).

2 Aggressive cases defined as those with stage III or IV and/or Gleason grade ≥8.

Analyses stratified by various indicators of oxidative stress (including smoking dose and duration, alcohol consumption, trial intervention assignment, fruit and vegetable intake, and dietary intake and/or blood levels of antioxidants such as carotenoids, vitamin E, and vitamin C) yielded significant interactions only for baseline serum levels of *β*-carotene. When we further stratified these results by trial intervention assignment (no *β*-carotene, *β*-carotene), the interactions between phytanic and pristanic acids and baseline circulating concentrations of *β*-carotene were only evident in the subgroup of men who were not randomized to the *β*-carotene intervention arm (Table 4). In this subgroup of participants, higher phytanic and pristanic acid levels were positively (yet not significantly) associated with risks of overall and aggressive (the latter for phytanic acid only) prostate cancer among men with lower circulating *β*-carotene levels, whereas these fatty acids were significantly inversely associated with risk among men with higher circulating *β*-carotene concentrations (all *P*-values for interaction <0.05). Baseline *β*-carotene concentrations were correlated with age, BMI, alcohol consumption, and smoking dose (Spearman correlation coefficients = 0.15, −0.15, −0.35, and −0.13; all *P*-values <0.05); however, adjustment for these variables in the aforementioned models did not alter any of the findings.

**Table 4:** OR and 95% CI for associations between phytanic and pristanic acids and prostate cancer risk stratified by baseline serum *β*-carotene concentrations, within trial intervention assignment groups.

	Serum *β*-carotene < 197 *μ*g/L		Serum *β*-carotene > 197 *μ*g/L	
	Cases/controls	Multivariate OR^1^ (95% CI)	Cases/controls	Multivariate OR^1^ (95% CI)
**Trial intervention assignment: NO*****β*****-carotene**				
*All cases*				
Phytanic acid tertiles				
1	29/36	1.0 (ref)	18/16	1.0 (ref)
2	26/21	1.53 (0.71–3.28)	27/21	1.04 (0.41–2.62)
3	22/18	1.54 (0.69–3.42)	21/45	0.40 (0.17–0.94)
*P*_trend_		0.24		0.02
*P*_interaction_ = 0.02				
Pristanic acid tertiles				
1	29/31	1.0 (ref)	25/22	1.0 (ref)
2	23/23	1.05 (0.48–2.30)	28/25	0.98 (0.44–2.17)
3	25/21	1.31 (0.60–2.83)	13/35	0.32 (0.13–0.76)
*P*_trend_		0.51		0.01
*P*_interaction_ = 0.03				
*Aggressive*^2^ *cases*				
Phytanic acid tertiles				
1	17/36	1.0 (ref)	11/16	1.0 (ref)
2	18/21	1.87 (0.79–4.43)	20/21	1.34 (0.49–3.67)
3	15/18	1.85 (0.75–4.57)	13/45	0.42 (0.16–1.13)
*P*_trend_		0.14		0.05
*P*_interaction_ = 0.02				
Pristanic acid tertiles				
1	17/31	1.0 (ref)	17/22	1.0 (ref)
2	13/23	1.03 (0.41–2.60)	20/25	1.03 (0.43–2.48)
3	20/21	0.83 (0.77–4.34)	7/35	0.26 (0.09–0.74)
*P*_trend_		0.18		0.01
*P*_interaction_ = 0.007				
**Trial intervention assignment: β-carotene**				
*All cases*				
Phytanic acid tertiles				
1	34/37	1.0 (ref)	17/11	1.0 (ref)
2	26/29	0.98 (0.48–2.03)	27/29	0.91 (0.37–2.24)
3	12/10	1.33 (0.51–3.49)	24/26	0.75 (0.30–1.91)
*P*_trend_		0.67		0.52
*P*_interaction_ = 0.42				
Pristanic acid tertiles				
1	33/31	1.0 (ref)	31/16	1.0 (ref)
2	21/23	0.84 (0.38–1.84)	28/29	0.50 (0.22–1.10)
3	18/22	0.79 (0.35–1.76)	26/21	0.64 (0.28–1.49)
*P*_trend_		0.54		0.30
*P*_interaction_ = 0.72				
*Aggressive*^2^ *cases*				
Phytanic acid tertiles				
1	19/37	1.0 (ref)	12/11	1.0 (ref)
2	16/29	1.10 (0.47–2.55)	25/29	0.80 (0.30–2.12)
3	9/10	1.78 (0.62–5.16)	24/26	0.85 (0.32–2.29)
*P*_trend_		0.36		0.80
*P*_interaction_ = 0.41				
Pristanic acid tertiles				
1	19/31	1.0 (ref)	21/16	1.0 (ref)
2	15/23	1.06 (0.43–2.59)	20/29	0.53 (0.22–1.25)
3	10/22	0.75 (0.29–1.91)	20/21	0.73 (0.30–1.79)
*P*_trend_		0.59		0.50
*P*_interaction_ = 0.94				

95% CI, 95% confidence interval; OR, odds ratio.

1 Unconditional logistic regression models adjusted for age and date of serum blood draw (matching factors).

2 Aggressive cases defined as those with stage III or IV and/or Gleason grade ≥8.

## Discussion

Excessive intake of branched-chain fatty acids that are metabolized by AMACR could provide an explanation for previously reported associations between dairy and red meat intake and prostate cancer risk. In this analysis, we found no statistically significant association between prediagnostic serum levels of phytanic or pristanic acids (derived predominantly from high-fat dairy products) and prostate cancer risk. Although we did observe that the relationship between these fatty acids and prostate cancer risk was dependent upon the subject's baseline *β*-carotene level, these subgroup findings are likely due to chance.

Two previous studies have evaluated circulating concentrations of phytanic acid in relation to prostate cancer risk, whereas none have examined pristanic acid in relation to this end point. The first was a small population-based case–control study in North Carolina that found serum phytanic acid levels were modestly, yet significantly, higher in prostate cancer cases compared to controls (0.10 vs. 0.08 mg/100 mL, *P*-value = 0.04) [18]. No point estimates or CI were provided in this report. The second study was a case–control analysis nested within the prospective European Investigation into Cancer and Nutrition Study, and involved 566 incident prostate cancer cases and an equal number of matched controls [19]. Higher plasma phytanic acid levels were associated with elevated risks of prostate cancer, but only in the subset of participants that had fasted for at least 3 h prior to blood draw. Our findings, which are based on fasting samples, differ in that we observed no increase in risk among men with elevated phytanic acid concentrations. Serum phytanic acid concentrations in our Finnish study population were generally much higher than in these previous studies, thus further complicating any direct comparison. Our findings also differ from a previous investigation of phytanic acid intake estimated using dietary questionnaire data in the ATBC cohort; in that study, higher intake of phytanic acid, as well as butter and cheese (the richest sources of this fatty acid), was associated with significant elevations in the risk of aggressive prostate cancer [23].

We evaluated potential effect modification of the phytanic–pristanic–prostate cancer associations by a variety of dietary and lifestyle indicators of oxidative stress. The only significant interactions that emerged were between baseline blood levels of *β*-carotene and the aforementioned fatty acids, and these interactions were only evident among the subgroup of men who did not receive trial *β*-carotene supplements during the ATBC trial. Given the large number of stratified analyses, no a priori rationale for this finding, and lack of similar interactions with other indicators of oxidative stress (including fruits and vegetables – the predominant food sources of *β*-carotene [29]), these findings are most likely due to chance. However, if the interactions are indeed real, they might be explained by the following: (1) phytanic and pristanic acids could have a stronger impact on the risk of prostate cancer among men with lower blood levels of *β*-carotene since peroxisomal degradation of these fatty acids generates free radicals [30], and *β*-carotene is a powerful antioxidant [29]; (2) phytanic and pristanic acids may have a protective effect on risk among men with higher blood levels of *β*-carotene because with sufficient antioxidant protection against the potentially prooxidative effects of these fatty acids (described above), higher levels might lead to altered (and beneficial) peroxisome proliferator-activated receptor (PPAR)-*α* and/or retinoid X receptor (RXR) signaling. There is in vitro evidence that phytanic acid is a ligand for PPAR-*α*—a regulator of lipid metabolism, cell proliferation, differentiation, adipogenesis, inflammatory signaling, and apoptosis that dimerizes with RXR before binding to response elements in target genes [31].

Strengths of our study include the use of fasting blood samples (since triacylglycerol-rich lipoproteins—in which phytanic acid is present—have been shown to increase in the circulation after consumption of meals containing fat [19]) that were collected prior to prostate cancer diagnosis, which minimizes reverse causation; availability of data on a wide range of potential confounders and effect modifiers; our ability to specifically evaluate associations with aggressive disease—which is arguably a more relevant clinical end point; and the use of a highly sensitive assay to measure circulating concentrations of phytanic and pristanic acids. A limitation was our reliance on fatty acids measured at a single point in time (at baseline), which increases the possibility of misclassification, especially given the long follow-up period. Studies with access to blood samples collected at multiple points in time are required in order to determine the stability of these biomarkers. However, it is reassuring that serum levels of both phytanic and pristanic acids were strongly correlated with a summary measure of phytanic acid intake that was created using food frequency data [28], as the latter instrument is intended to capture usual intake over a long period of time [32]. Given the potentially large degree of measurement error in food composition data for phytanic acid, it is likely that the true correlation between phytanic acid intake and its corresponding biochemical measure would be even stronger. Another possible weakness is our measurement of phytanic and pristanic acids in serum rather than erythrocytes; in general, erythrocyte fatty acids better reflect long-term intake as they are less sensitive to recent diet and have longer half-lives than plasma or serum fatty acids [33]. Additional potential weaknesses include the high correlation between phytanic and pristanic acids and dairy fat, which precludes examination of independent effects, as well as the nature of the ATBC cohort (older male smokers), which may limit the generalizability of our findings to other populations. Finally, ascertainment bias may have been present as cases in our nested sample were more highly educated and appeared to lead a healthier lifestyle (significantly higher serum *β*-carotene levels, significantly lower number of years smoked, and marginally leaner and more physically active) than controls, which could reflect increased access to and utilization of healthcare services. However, cases did not appear to be different from controls with respect to dietary factors, which are typically indicative of lifestyle behaviors.

In summary, we found no overall relationship between prostate cancer and blood levels of phytanic and pristanic acids—fatty acids derived predominantly from high-fat dairy products. Further exploration of these associations is required in racially diverse populations and in nonsmokers. Investigation of potential interactions between phytanic and pristanic acids and common genetic variants in the branched-chain fatty acid metabolism pathway (particularly AMACR [16]) with respect to prostate cancer may also be informative. Finally, all men should adhere to the American Cancer Society guidelines on nutrition and physical activity for cancer prevention, which emphasize maintaining a healthy weight, being physically active, eating a healthy diet with an emphasis on plant-based foods, and limiting alcohol intake [34].

## Conflict of Interest

None declared.
